# Incretin hormones as immunomodulators of atherosclerosis

**DOI:** 10.3389/fendo.2012.00112

**Published:** 2012-09-07

**Authors:** Nuria Alonso, M. Teresa Julián, Manuel Puig-Domingo, Marta Vives-Pi

**Affiliations:** ^1^Endocrinology and Nutrition Department, Hospital Universitari Germans Trias i PujolBadalona, Spain; ^2^Laboratory of Immunobiology for Research and Application to Diagnosis, Blood and Tissue Bank, Institute Germans Trias i PujolBadalona, Spain

**Keywords:** atherosclerosis, diabetes, GLP-1, incretins, GIP

## Abstract

Atherosclerosis results from endothelial cell dysfunction and inflammatory processes affecting both macro- and microvasculature which are involved in vascular diabetic complications. Glucagon-like peptide-1 (GLP-1) is an incretin hormone responsible for amplification of insulin secretion when nutrients are given orally as opposed to intravenously and it retains its insulinotropic activity in patients with type 2 diabetes mellitus (T2D). GLP-1 based therapies, such as GLP-1 receptor (GLP-1R) agonists and inhibitors of dipeptidyl peptidase-4, an enzyme that degrades endogenous GLP-1 are routinely used to treat patients with T2D. Recent experimental model studies have established that GLP-1R mRNA is widely expressed in several immune cells. Moreover, its activation contributes to the regulation of both thymocyte and peripheral T cells proliferation and is involved in the maintenance of peripheral regulatory T cells. GLP-1R is also expressed in endothelial and smooth muscle cells. The effect of incretin hormones on atherosclerogenesis have recently been studied in animal models of apolipoprotein E-deficient mice (apoE^-/-^). These studies have demonstrated that treatment with incretin hormones or related compounds suppresses the progression of atherosclerosis and macrophage infiltration in the arterial wall as well as a marked anti-oxidative and anti-inflammatory effect on endothelial cells. This effect may have a major impact on the attenuation of atherosclerosis and may help in the design of new therapies for cardiovascular disease in patients with type 2 diabetes.

## INTRODUCTION

Diabetes is global health problem with a prevalence of more than 285 million cases worldwide and an incidence that continues to increase. The vast majority of diabetic patients (~90–95%) suffer from type 2 diabetes (T2D), whereas type 1 diabetes (T1D), accounts for 5–10% and rare forms (i.e., genetic forms of diabetes, diabetes secondary to pancreatic diseases or surgery, as well as gestational diabetes) constitute the remaining subtypes (International Diabetes Federation, 2009). Cardiovascular complications represent the primary source of morbidity and mortality in diabetic subjects (Mazzone et al., 2008) and it is well known that diabetic milieu *per se* accelerates the course of atherosclerosis (Nogi et al., 2012). It is also well established that T2D is caused by a combination of insulin resistance in skeletal muscle, liver, and adipose tissues and impaired insulin secretion from the pancreatic islets (Stumvoll et al., 2005). Insulin resistance is the main feature of metabolic syndrome, which refers to the clustering of cardiovascular risk factors that include diabetes, obesity, dyslipidemia, and hypertension (Bajaj and Defronzo, 2003). In relation to insulin resistance, the mechanisms that can promote both atherogenesis and advanced plaque progression likely involve both systemic factors that promote these processes, particularly dyslipidemia, but also hypertension and a proinflammatory state as well as the effect of perturbed insulin signaling at the level of the intimal cells that participate in atherosclerosis (Bornfeldt and Tabas, 2011). There is extensive evidence indicating that insulin resistance increases the risk of coronary artery disease (CAD) even in the absence of hyperglycemia (DeFronzo, 2010). *In vivo* studies have provided data showing that insulin resistance in macrophages and endothelial cells may promote atherogenesis and clinical progression of advanced plaques (Rask-Madsen et al., 2010). Data from human and animal studies supporting a direct pro-atherogenic role of hyperglycemia in vascular cells are not as strong as for insulin resistance but there is suggestive evidence that high glucose is atherogenic, particularly at the level of the arterial endothelium by promoting early stages of lesion formation (Vikramadithyan et al., 2005). However, it is possible that hyperglycemia acts also synergistically with other cardiovascular risk factors and even insulin resistance itself in advanced lesions in the setting of T2D (Bornfeldt and Tabas, 2011). For example, glucotoxicity may contribute to insulin resistance, and treatment of hyperglycemia in T2D improves insulin resistance in some tissues (Henry, 1996).

Two novel classes of glucose-lowering agents for the treatment of T2D have been introduced in the market in the last years: glucagon-like peptide-1 receptor (GLP-1R) agonists and dipeptidyl peptidase-4 (DPP-4) inhibitors or incretin enhancers (Tahrani et al., 2011). The mechanism of action of these drugs is based on the enhancement of the incretin effect. The well-documented phenomenon of oral glucose eliciting a higher insulin response than intravenous glucose at identical plasma levels of glucose is known as the incretin effect (McIntyre et al., 1964). The incretin effect has been found to be mediated mainly by two gut-derived hormones: glucagon-like peptide-1 (GLP-1) and glucose-dependent insulinotropic polypeptide (GIP; Baggio and Drucker, 2007). These incretins control blood glucose by stimulating insulin release from the β cells of the pancreatic islet, decreasing glucagon secretion and slowing gastric emptying (Nauck et al., 2011). However, both GLP-1 and GIP are rapidly inactivated *in vivo* by circulating peptidases, mainly DPP-4/CD26. Thus, the administration of DPP-4 inhibitors prevents the degradation and inactivation of GLP-1 and GIP. In addition to their antidiabetic action through the aforementioned mechanisms, recent experimental and clinical studies have demonstrated that incretin therapies have several effects on cardiovascular function (Grieve et al., 2009). These effects are possibly mediated at least in part by mechanisms independent of their glucose-lowering activity and include: changes in blood pressure (Brown, 2012), endothelial function (Irace et al., 2012), body weight (Vilsbøll et al. 2012), cardiac metabolism (Nielsen et al., 2012), lipid metabolism (Farr and Adeli, 2012), left ventricular function (Poornima et al., 2008), and the response to ischemia-reperfusion injury (Chinda et al., 2012).

It is now widely accepted that inflammation and immunity play important roles in the pathogenesis of atherosclerosis (Hansson and Libby, 2006). Recently, several studies have shown that DPP-4 inhibitors and GLP-1R agonists exert also a potent anti-inflammatory effect and thus, may potentially contribute to the prevention of atherosclerosis (Chaudhuri et al., 2012; Makdissi et al., 2012).

## ATHEROSCLEROSIS AS AN IMMUNE-MEDIATED DISORDER

Studies over the last decade strongly support the idea that atherosclerosis results from endothelial cell dysfunction followed by lipid accumulation and an inflammatory process affecting both macro- and microvasculature (Lusis, 2000). But also, atherosclerosis is now universally recognized as having an inflammatory immune-mediated component in disease development in which both the monocyte and the macrophage are central to this inflammation (Ross, 1999). Macrophage infiltration is one of the driving factors for plaque development and during the first stages of atherosclerosis, they are actively recruited from the circulation and the *vasa vasorum* into the intimal lining of blood vessels. On the other hand, there is now solid evidence that T cell are involved in atherogenesis. The available data suggest that T cell-mediated responses contribute to both the development and the progression of atherosclerosis. The majority of pathogenic T cells involved in atherosclerosis are of Th1 profile producing high levels of INF-γ among other cytokines which are known to activate macrophages and dendritic cells, leading to the perpetuation of this pathogenic Th1 response (Hansson, 2001). In addition, IFN-γ may inhibit vascular smooth muscle cell (SMC) proliferation and reduces local collagen production. Matrix metalloproteinases are also up-regulated, thereby contributing to the thinning of the fibrous cap (Tedgui and Mallat, 2006). Deficiency of IFN-γ receptor or IFN-γ significantly reduces lesion development and enhances collagen content of the plaque, whereas exogenous administration of IFN-γ stimulates lesion development (Whitman et al., 2000).

In relation to Th2 cells, Th2-biased responses have been proposed to antagonize pro-atherogenic Th1 effects and thereby confer atheroprotection. However, the role of Th2 pathway in the development of atherosclerosis remains controversial depending on the stage and/or site of the lesion, as well as on the experimental model used (Mallat et al., 2009). In mouse models that are relatively resistant to atherosclerosis, a Th2-bias has been shown to protect against early fatty streak development (Huber et al., 2001). However, in other models using LDLR^-/-^ mice, deficiency in IL-4, the prototypic Th2-related cytokine, had no substantial effect on lesion development al least in one study (King et al., 2007). However, while initial studies focused more on the pathogenic arm of the immune system, recent work clearly suggests an important role for several subsets of regulatory T cells (Treg) in the protection against lesion development (Wigren et al., 2011). These cells home to peripheral tissues to maintain self-tolerance and prevent autoimmunity by inhibiting pathogenic lymphocytes (Sakaguchi et al., 2009). Data gathered from the literature indicate that several populations of Tregs “tune down” the inflammatory response within the atherosclerotic lesion in transgenic atherosclerosis-prone mice which points to a protective role of Tregs in this process (Mallat et al., 2003).

## INCRETIN HORMONES AND ATHEROSCLEROSIS

Two main incretin hormones have been fully characterized to date: GLP-1 and GIP. GLP-1 stimulates insulin and inhibits glucagon secretion in a glucose-dependent manner. It also inhibits gastric emptying and reduces appetite, actions that contribute to improved glycemic control in T2D patients (Kazakos, 2011). It is synthesized and secreted by enteroendocrine L cells distributed through the small and large intestine; however, the majority of intestinal GLP-1 content has been localized to the distal small bowel and colon (Brubaker, 2010). The GLP-1R was originally identified in islet β cells but is widely expressed in extrapancreatic tissues, including the lung, kidney, central nervous system, enteric and peripheral nervous system, lymphocytes, macrophages (Bullock et al., 1996), human coronary endothelial cells (Erdogdu et al., 2012), human umbilical vein endothelial cells (HUVECs; Ding and Zhang, 2012), and heart (Wei and Mojsov, 1995). GIP is synthesized in and secreted from enteroendocrine K cells localized to the proximal small bowel (Ussher and Drucker, 2012). GIP receptor (GIP-R) are widely expressed in extrapancreatic tissues, including the gastrointestinal tract, adipose tissue, heart, pituitary, adrenal cortex, and multiple regions of the central nervous system (Usdin et al., 1993).

The mechanisms by which incretin modulation might be associated with cardiovascular benefits are multiple. Considerable evidence incriminates the dysfunctional adipocyte and excess ectopic adiposity – in the liver, around visceral organs, and/or in the skeletal musculature – as culprits in both T2D and its atherosclerotic complications (Bays, 2011). Adipose tissue is an important inflammatory source in obesity and T2D, not only because of cytokines produced from the adipocyte itself, but also because of infiltration by proinflammatory macrophages (Shoelson and Goldfine, 2009). Thus, weight loss associated with GLP-1R agonists decreases cardiovascular risk in successfully treated subjects and may have a positive influence in cardiovascular endpoints. In relation to lipid metabolism, a direct role for GLP-1 in the control of chylomicron secretion has been suggested. Indeed, treatment with GLP-1 as well as GLP-1R agonists in animals models reduces triacylglycerol (TAG) absorption, decreases intestinal lymph flow, and reduces intestinal B-48 apolipoprotein production (Qin et al., 2005). In T2D patients, treatments with GLP-1R agonists and DPP-4 inhibitors have demonstrated favorable effects on postprandial dyslipidemia (TAG, Apo B-48, and FFA; Hsieh et al., 2010).

**FIGURE 1:**
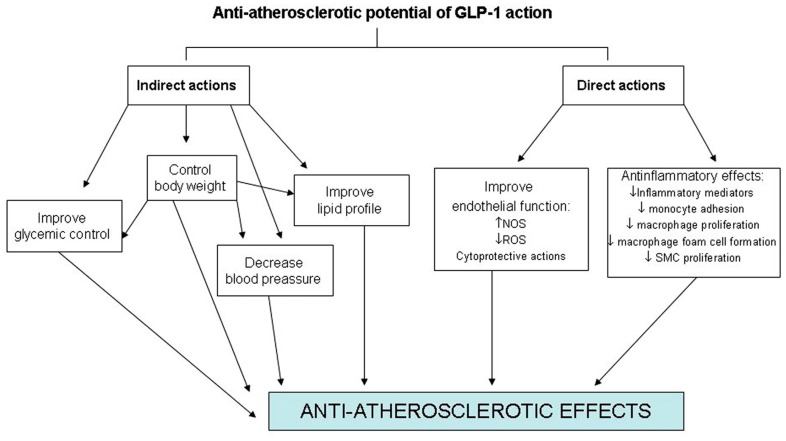
**Direct and indirect effects of GLP-1 on the cardiovascular system**.

On the other hand, GLP-1R expression in endothelial cells, vascular SMCs, monocytes, macrophages, and lymphocytes also raises the prospect for direct effects on atherosclerosis and inflammation (**Figure 1**). However, little information exists on the effects of GLP-1 on atherogenesis itself. Elevated tumor necrosis factor-alpha (TNF-α) levels and hyperglycemia are implicated in diabetes-associated endothelial cell dysfunction and may be causal in premature atherosclerosis (Iwasaki et al., 2008). Indeed, TNF-α and hyperglycemia have been shown to induce plasminogen activator inhibitor-1 (PAI-1) and vascular cell adhesion molecules (VCAM-1 and ICAM-1) expression in human vascular endothelial cells (Morigi et al., 1998). Recently, the protective properties in endothelial cells of GLP-1 and liraglutide, a GLP-1R agonist, have been demonstrated by several investigators. This treatment reduces TNF-α-mediated expression of PAI-1, ICAM-1, and VCAM-1 expression in human vascular endothelial cells (Liu et al., 2009) as well as TNF-α induced oxidative stress (Shiraki et al., 2012). In relation to the atherosclerotic lesion, recent experimental studies have shown that GLP-1R activation by the administration of exendin-4 significantly reduces the accumulation of monocytes/macrophages in the vascular wall of C57BL/6 and ApoE^-/-^ mice. This effect seems to be mediated, at least in part, by suppressing the inflammatory response in macrophages through the activation of the cAMP/PKa pathway which inhibits the expression of TNF-α and the monocyte chemoattractant protein-1 (MCP-1) in activated macrophages (Arakawa et al., 2010). Administration of active forms of native incretins (GLP-1 and GIP) has also been described to be associated with a suppression of atherosclerotic lesions and macrophage infiltration in the vascular wall in ApoE^-/-^ mice. This treatment directly suppresses the expression of MCP-1, VCAM-1, ICAM-1, and PAI-1 in aortic endothelial cells, as well as suppresses aortic SMCs proliferation and macrophage foam cell formation associated with the down-regulation of CD36, a type A scavenger receptor that takes up modified LDLs, and acyl-coenzyme A: cholesterol acyltransferase-1 (ACAT-1), the enzyme that promotes cholesteryl ester accumulation in macrophages (Nagashima et al., 2011).

Glucagon-like peptide-1 receptor protein expression is not only found in endothelial cells and macrophages but also in murine SMCs suggesting that GLP-1R agonists may have direct effects on SMCs during neointimal formation. The effects of exendin-4, a GLP-1R agonist, on intimal thickening after vascular injury have been investigated in a recent study. The results obtained have shown that treatment with a GLP-1R agonist is associated with a reduced intimal thickening after vascular injury. This effect seems to be mediated through a reduced proliferation of SMC induced by platelet-derived growth factor (PDGF) which seems to be independent of the canonical (cAMP) GLP-1R signal pathway suggesting a direct action of GLP-1 on SMCs (Goto et al., 2011).

The truncated metabolite of GLP-1 (9–36) amide has also been described to exert some cardioprotective effects, increasing basal myocardial glucose uptake, and improving left ventricular function in animal models with cardiomyopathy (Nikolaidis et al., 2005; Ban et al., 2008).

Since its description in 1966, DPP-4 has been considered to be a unique peptidase that cleaves dipeptides from peptides and proteins containing proline in the penultimate position. However, proteolysis is only one of the multiple functions that this protein executes. Other functions attributed to this glycoprotein include the regulation of T cell activation, DNA synthesis, cell proliferation, cytokine production, and signaling activation (Hegen et al., 1997). In addition to the action of CD26/DPP-4 on GLP-1 and GIP, CD26/DDP-4 directly activates a number of proteins such as mitogen-activated protein kinases (MAPKs) which are involved, in particular the extracellular signal-regulated kinase (ERK), in cell proliferation. In a recent *in vitro* study it has been demonstrated that the inhibition of DPP-4/CD26 by alogliptin suppresses Toll-like receptor (TLR)-4-mediated ERK activation and ERK-dependent matrix metalloproteinases expression by histiocytes (Ta et al., 2010). The ERK pathway is an important signaling cascade involved in many physiological and pathophysiological processes including cell proliferation, apoptosis, angiogenesis, and inflammation (McCubrey et al., 2007). Thus, results of this study suggest that DPP-4/CD26 may play an important role in macrophage-mediated inflammation response and tissue remodeling since matrix metalloproteinases are crucially involved in atherosclerosis. The inhibition of DPP-4 has also demonstrated a reduction of atherosclerotic lesions in diabetic apolipoprotein E-deficient mice. Moreover, *ex vivo* studies have shown that DPP-4 inhibition attenuates diabetes-augmented IL-6 and IL-1β expression in atherosclerotic plaques (Ta et al., 2011) and reduces plaque macrophages infiltration and monocyte migration to the aorta of male LDLR^-/-^ mice (Shah et al., 2011).

Two recent clinical studies have evaluated the anti-inflammatory effect of a GLP-1R analog (exenatide) and a DPP-4 inhibitor (sitagliptin) in a group of patients with T2D. Results of these studies have demonstrated that both treatments have a potent and rapid anti-inflammatory effect with a significant reduction in reactive oxygen species generation and the mRNA expression of several inflammatory mediators (TNF-α, JNK-1, TLR-2, TLR-4, IL-1β, and SOCS-3) in mononuclear cells, which might potentially contribute to the inhibition of atherosclerosis. Remarkably, these anti-inflammatory effects occurred at an earlier phase of treatment and were independent of weight loss (Chaudhuri et al., 2012; Makdissi et al., 2012).

On the other hand, DPP-4 or CD26 cleaves other multiple peptide substrates, many of which have direct actions on the heart and blood vessels. Among them are included the stromal cell-derived factor-1α (SDF-1α), the neuropeptide Y (NPY), the peptide Y (PYY), the B-type (brain) natriuretic peptide (BNP), and the GLP-2 (Ussher and Drucker, 2012). In relation to SDF-1α, a chemokine that promotes homing of endothelial progenitor cells to sites of cellular injury, considerable evidence supports a role for SDF-1α as a cardioactive DPP-4 substrate. Indeed, DPP-4 inhibitors have been used, mainly in combination with granulocyte-colony-stimulating factor (G-CSF), to increase stem cell number in both preclinical and clinical studies of cardiovascular injury (Zaruba et al., 2009). The therapeutic use of SDF-1 has also been studied in an animal model to treat the peripheral artery disease. The study shows that SDF-1 engineered to be resistant to DPP-4 improves the blood flow (Segers et al., 2011).

Little has been published on GIP and atherosclerosis. GIP has a potent stimulatory effect on insulin release from the pancreas, but several experimental studies have demonstrated that GIP looses this action in diabetes because GIP-R in pancreatic islets are substantially down-regulated in a hyperglycemic state (Lynn et al., 2001, 2003). This may explain the inability of GIP to induce insulin secretion in diabetes. Recently, Nogi et al. (2012) have described that chronic administration of GIP at a level several-fold higher remarkably suppresses the progression of atherosclerosis in STZ-induced diabetic ApoE^-/-^ mice. In this study, GIP infusion significantly suppressed macrophage-driven atherosclerotic lesions and reduced foam cell formation in macrophages, even though GIP-R expression in macrophages was partially down-regulated in the diabetic state. Recently a published experimental study suggests that GIP could block the signal pathways of advanced glycation end products (AGEs) in HUVECs, which play a crucial role in vascular damage in diabetes (Ojima et al., 2012). The same protective mechanism has been described with GLP-1 (Zhan et al., 2012).

Finally, the majority of GLP-1R agonists and DPP-4 inhibitors are undergoing assessment of cardiovascular outcomes in large, multicenter clinical trials of cardiovascular outcomes. To date, results of randomized trials do not suggest any detrimental effect of GLP-1 receptor agonists on cardiovascular events. However, specifically designed longer-term trials are needed for verifying the possibility of a beneficial effect (Monami et al., 2011a). In relation to DPP-4 inhibitors a recent meta-analysis suggests a possible protection from cardiovascular events, although results should be interpreted with caution, as those events were not the primary endpoint, the trial duration was short, and the characteristics of patients included could be different from routine clinical practice (Monami et al., 2011b).

## CONCLUSION

In conclusion, there is now overwhelming evidence that the macrophage has a crucial role in the initiation and progression of atherosclerotic plaque and thus has emerged as a novel therapeutic target for the treatment of atherosclerosis. Recent studies suggest that incretin agents seem to have direct effects on macrophages and endothelial cells which are both involved in the progression of atherosclerosis. Despite the intriguing findings in animals, data on the long-term effects of incretin-based therapy on atherosclerosis-associated outcomes in diabetic humans are not yet available. The critical issue of whether the anti-atherogenic action of incretin agents can be translated into improved cardiovascular outcomes for diabetic patients remains to be elucidated with prospective, large-scale clinical trials.

## Conflict of Interest Statement

The authors declare that the research was conducted in the absence of any commercial or financial relationships that could be construed as a potential conflict of interest.
